# The impact of temporary contracts on suicide rates

**DOI:** 10.1371/journal.pone.0252077

**Published:** 2021-05-26

**Authors:** Ricard Grèbol Jiménez, Judit Vall Castelló

**Affiliations:** 1 Department of Economics, University Carlos III Madrid (UC3M), Barcelona, Spain; 2 Department of Economics, University of Barcelona (UB), Barcelona Institute of Economics (IEB) and CRES-UPF, Barcelona, Spain; University of Luxembourg and Luxembourg Institute of Socio-Economic Research (LISER), LUXEMBOURG

## Abstract

The number of suicides has increased in the last decades in several developed countries. For instance, Spain has experienced a gradual but steady increase in suicides since the 80’s and it is currently the leading external cause of death in the country. At the same time, the dualisation of the labor market, with a strong and persistent incidence of temporary contracts, has increased the instability of employment conditions. Both developments have a stronger incidence for individuals with lower levels of education. Therefore, in this paper we use rich administrative data in order to estimate the impact of the wide spread use of temporary contracts on suicides. In order to do that we exploit a reform that liberalised the use of fix-term contracts in Spain in 1984. Our results show strong long-term effects of the reform, which increased the suicide mortality rate of affected cohorts (those entering the labor market just after the liberalisation) by at least 25.3%. We believe that this result has important policy implications and should be taken into account in the design of the national suicide prevention plans.

## 1 Introduction

Suicide is a global phenomenon and the leading external cause of death in high-income countries [1]. While the association between unemployment and suicide has been widely studied [2–7], the role of employment protection legislation (EPL), the set of regulations limiting the accessibility of firms to hire and fire employees, on suicidal behaviour is so far an open question. Addressing this issue is of particular importance for typical high EPL European countries due to their high age-adjusted suicide rates compared to other Regions [8]. As a matter of fact, the European Region has the second-highest age-standardized suicide rates across WHO regions (12.9 per 100.000 individuals) and the undoubtedly highest one for males (21.2 per 100.000 individuals).

A limitation of existing literature is that it uses a dichotomous analysis of unemployment not reflecting the dynamics of employment instability. This type of analysis does not deal with the complexity of employment conditions and only identifies part of the labour market instability affecting suicidal behaviour. Job insecurity cannot be limited to the unemployment condition and has to be considered as a many-sided notion that includes the quality of employment, economic conditions, type of contracts, job sector, among others concepts. Caroli and Godard (2016) [9] propose an instrumentation of perceived job insecurity by the stringency of the EPL and find that unstable employment has a negative impact on several health outcomes. Evaluating the impact of EPL on mental health outcomes and suicide rates is a present-day policy-relevant issue because of the seriousness of this phenomenon in the working-age population and the steady increase of job insecurity perception in high-income countries [10].

Throughout the last decades, many European countries have implemented downsizing reforms in their EPL systems, promoting flexibilisation of the labour market through fixed-term contracts. As a consequence of such reforms, labour market segmentation has arisen, whereby some workers enjoy lower employment stability, lack of career development, and low rates of upgrading from temporary contracts to permanent ones [11], while others enjoy high protection and stability. Empirical evidence on the impact of fixed-term contracts or temporary employment on labour market outcomes is mixed. Previous research findings suggest a positive short-run effect but a negative long-run impact of the number of days worked and earnings of less educated workers. For Spain, García-Pérez et al. (2019) [12] find that a reform that liberalized the use of fixed-term contracts increases the overall number of temporary contracts and reduces the number of days in employment. Consequently, the yearly earnings losses for less educated workers entering the labour market just after the implementation of the reform amounts to 7.3% in the long run, compared to workers entering the labour market before the reform.

In our paper, we study the long-term impact of the liberalization of fixed-term contracts on suicide mortality rates. We believe that this represents an important contribution to the literature as well as in policy terms given the current limitations to understanding the link between working conditions and suicidal behaviour. More specifically, we evaluate the 1984 Spanish Labour Market Reform which liberalised the use of fixed-term contracts. While, compared to other high income countries, Spain has a relatively lower age-standardized suicide mortality rates, it represents an ideal setting to evaluate the impact of the reform in the long run due to its early liberalisation of fixed-term contracts [13]. After the Spanish experience, other European countries have implemented similar labour market reforms which reinforces the external validity and the generalisability of our findings.

To shed light on the issue, we use the restricted access version of the *Deaths statistics* from the Spanish Statistical Office (INE) to track cohorts of individuals before and after the reform. We do the analysis by gender and education, particularly focusing on three different levels of education: high school dropouts, high school first-level graduates, and high school second-level graduates. As the minimum legal working age in Spain is 16 years, we assign the labour market entry of high school dropouts to be 16 and, for the other levels of educational attainment we assign the graduation date as the labour market entry age.

One of the main methodological challenges when studying suicides is the low base rate of suicide and the complexity and variety of the channels that are compatible with this outcome [14]. Therefore, as our identification strategy we use a quarter cohort regression discontinuity design [15] around the cohorts entering the labour market one trimester before and one trimester after the implementation of the reform. We perform this analysis for suicides between 2014 and 2018 as INE started providing the educational attainment level in the *Deaths statistics according to cause of deaths* dataset, under restricted access conditions. Although existing literature finds a link between macroeconomic economic conditions and mental health outcomes and suicide rates [16–18], our treated and control groups only differ in one trimester of birth and are, therefore, exposed to the same economic conditions at, practically, the same age.

Several identified risk factors for suicides relate to medical, psychosocial, cultural, genetic, and socioeconomic conditions [19–21]. Their significance differs by gender, and adverse labour market outcomes are associated with higher suicide risk in men [22–23]. In our analysis, we find empirical evidence that less educated male workers who entered the labour market in the trimester just after the reform have a larger probability of committing suicide in the long run than those entering just before the reform. Our results show that the reform increases the number of high school first-level male graduates commiting suicide by at least 25.3%. We find no significant effect of the reform on women or high-school second-level graduates. These results are consistent with prior literature showing that employment instability particularly affects men health outcomes and that the liberalisation of fixed-term contracts had no significant effect on the working conditions of higher educated workers. However, we do not find any significant effect for high-school dropouts, suggesting some unspecified pathways through which fixed-term contracts may affect suicide rates.

Ideally, we would have data for the whole period and would be able to use a duration model of the suicide mortality hazard rate by our different education and gender subgroups for a complete analysis. The main limitation of our analysis is that while we find evidence on the suicide rates for a subgroup of less educated males in the long run, it is likely that other subgroups such as dropouts males suffered the impact of the reform on their likelihood to commit suicide in other periods, such as during economic recessions or during the COVID-19 pandemic. Another possible hypothesis is that the difference in the impact of the reform between high-school dropouts and first-level graduates is due to social priorities across both groups. Phillips and Hempstead (2017) [24] find that relationship problems and drugs addiction are common circumstances for less educated individuals committing suicide. Also, mental health issues and career problems are more prevalent among higher educated workers. This may suggest different types of mechanisms among educational attainment groups due to dissimilarities in priorities, job sectors, or location. However, as we have no information on the cause of suicide or the job sector of deceased individuals, we cannot corroborate the validity of theses hypotheses. Our findings suggest that determining these manifold contributing factors is crucial to disentangle the heterogeneity effects of the impact of the liberalisation of fixed-term contracts on suicide rates and a question of high policy relevance for suicide prevention.

Moreover, we also report the long-term impact on drug overdose mortality and find also a significant effect of the reform for male high school first-level graduates. This further suggests that the liberalisation in the regulation of fixed-term contracts and the channels through which the reform affects suicides have also an impact on other health outcomes.

The key identification assumption used in our RD design is the continuity around both sides of the reform cutoff point. Previous concerns on this issue for labour market outcomes were addressed using a difference-in-differences specification by García-Pérez et al. (2019) [12]. The requirement that the only change at the discontinuity having an impact on suicides is the reform is credible in this particular setting as shown by the graphical analysis in Section 3. García-Pérez et al. (2019) [12] show the continuity of the running variable by education, particularly that our studied reform did not have an effect on the education decision. Additionally, we assess the continuity assumption through two placebo tests. The first one uses a falsification test simulating the reform implementation on an earlier period and the second uses an alternative outcome as a placebo, external causes of death due to accidents, which should be unaffected by the reform.

Our paper makes, at least, two contributions. First, despite previous literature evaluating the role of job insecurity and employment protection on self-assessed health and health disorders, as far as we are aware of, this is the first paper identifying the causal impact of employment stability and the liberalisation of fixed-term contracts on cause-specific mortality. Some of the existing literature exploring the role of temporary employment on mortality [25] or the association between employment protection and suicides [26] use non-experimental techniques and non-causal estimates for identification. Instead, we use a cohort RDD that exploits a large labour reform in Spain and we can identify the causal impact of the liberalisation in the regulation of fixed-term contracts on suicides. Moreover, most of the previous literature examines the role of unemployment on suicide rates whereas, in our paper, we estimate the impact of EPL, aiming at better capturing perceived job insecurity.

The second contribution of the study is related to the timing of the analysis as our focus is on the long-term impacts of the reform. While there is previous literature finding a negative impact of fixed-term contracts on labour market outcomes in the long run, our results show the first evidence of the long-term impact of fixed-term contracts on suicide rates. This is an important contribution in terms of policy implications given the recent reforms in European EPL systems and the widespread use of fixed-term contracts in several countries. Understanding the contributing factors to suicides is essential for suicide prevention policies and legislation. Furthermore, examining long-run outcomes allows us to uncover the undocumented effects of fixed-term contracts on health outcomes.

However, this paper has some limitations due to data availability. First,we are not able to explore the impact of fixed-term contracts on suicides in the short-run and for different age groups. This is a relevant question to address as suicide is one of the leading causes of death among young people. Second, our estimations could only evaluate a period of economic growth whereas the impact during an economic recession is left to be studied. An extensive literature evaluating the effects of negative macroeconomic conditions and unemployment find these periods are associated with an increase of completed suicides. Therefore, evaluating the impact of fixed-term contracts on other economic conditions is important as the effect of fixed-term contracts will likely be larger during economic stress periods. Third, further research should develop heterogeneity effects of the impact of the fixed-term contracts on suicides. For instance, differences in family structures, job sectors, or locations (e.g., urban, suburban and rural areas) are likely to establish distinct suicide rate outcomes.

The rest of the paper is organised as follows. Section 2 outlines the characteristics of the labour reform and the suicide situation in Spain. Section 3 describes the data and the empirical methodology. Empirical results are reported and discussed in Section 4. Section 5 presents some robustness checks and extensions, and Section 6 concludes.

## 2 Temporary contracts and suicide evolution in Spain

### 2.1 Spanish EPL and fixed-term contracts liberalisation

The Workers’ Statute introduced in Spain in 1980 designed temporary contracts to be used only for temporary reasons such as seasonal jobs. These restrictions on temporary contracts meant that they were rarely used in the early-1980s. However, aiming job creation due to high unemployment rates, the Spanish government implemented a labour liberalisation reform in 1984. The reform regulating the temporary contracts was passed in August 1984 but was implemented on October 17th by the Real Decreto 1989/1984 published in the BOE (Spanish Official State Bulletin). It consisted of the liberalisation of temporary contracts and a reduction of dismissal costs. This meant that temporary contracts were no longer required to be only used for temporary reasons but could be signed for all activities and their duration was limited between a minimum of six months and a maximum of three years. Moreover, fixed-term contracts had low dismissal severance payments compared to permanent contracts. On one hand, firing costs at termination were 8 days per salary per year of job tenure in the case of temporary contracts. On the other hand, permanent contracts severance payments depended on whether the cause of dismissal was considered fair or unfair. Dismissal was only considered as fair if the firm was able to argue that the employee was not capable of performing the job tasks or if it was required due to economic or technological reasons. While unfair cost firing was equal to 45 days of salary per year of job tenure with a maximum of 42 months of wages, for fair dismissals cost firings amount to 20 days with a maximum of one year’s wages. As the 1984 labour reform did not modify permanent contracts, incentives for firms to employ fixed-term contracts increased and there was a strong substitution of permanent by temporary workers [27].

Temporary contracts represented a low percentage of the total number of non-agricultural private sector employees until the labour reform was passed on the last trimester of 1984 [28] After the reform, the percentage of temporary employees over total employment increased over time, especially for young workers, and Spain reached soon the first position in the proportion of temporary contracts among OECD countries. For instance, the percentage of temporary employment in Spain was 32.2% in 2000, while the EU (28 countries, UK included) average was 12.7%. Temporary employment rates are particularly high among young workers and were already around 70% in the early-1990s. Moreover, temporary contracts were widely adopted very soon after the reform, and their use heavily affected young employees.

In a recent publication, García-Pérez et al. [12] argue that the Spanish 1984 labour reform and the liberalisation in the regulation of fixed-term contracts had a negative long-term impact on the number of days worked and earnings of less educated workers. They find evidence that workers entering the labour market just after the implementation of the reform had a higher chance of having a fixed-term contract than workers entering the labour market a trimester before, and this enhanced a vicious chaining cycle of temporary jobs without upgrading to a permanent one. While the impact was larger in the first 10 years of these workers’ careers, the yearly earning losses after 27 years on less educated workers still amount to 7.3% due to the liberalisation of fixed-term contracts. At the same time that fixed-term contracts enhanced less educated young workers to land a first job, it worsened their career paths. The negative long-term effects of the liberalisation of fixed-term contracts are not limited to labour outcomes but other negative side effects, such as an impact on suicide rates of less educated workers, are present.

Therefore, to estimate these long-term effects we use a RDD strategy comparing individuals who reach the labour market entry age of 16, or in their graduation date if required, before and after the reform. This empirical strategy allows us to evaluate the long-term impact of the Spanish 1984 labour reform on completed suicides for different educational attainment level groups. Our paper aims to understand whether entering the labour market under different situations (wide availability or not of temporary contracts) increases long-term vulnerability to suicidal behaviour.

### 2.2 Suicide in Spain

Premature death from suicide in Spain is the current leading external cause of death and responsible for more than twice the traffic accidents deaths. According to OECD latest statistics from 2017, Spain has a yearly suicide rate of 7.0 suicides per 100.000 persons. However, considerable gender differences are common in suicides, and suicides rates in Spain reach 10.9 suicides per 100.000 men while falling to 3.6 for women. Suicide rates differ considerably across developed countries and Spain has a relatively low number of suicides compared to other OECD countries. For instance, the United States has a yearly suicide rate of 14.0 suicides per 100.000 persons, France has a suicide rate of 12.3, and Belgium of 15.9 suicides per 100.000 persons.

**Fig 1:**
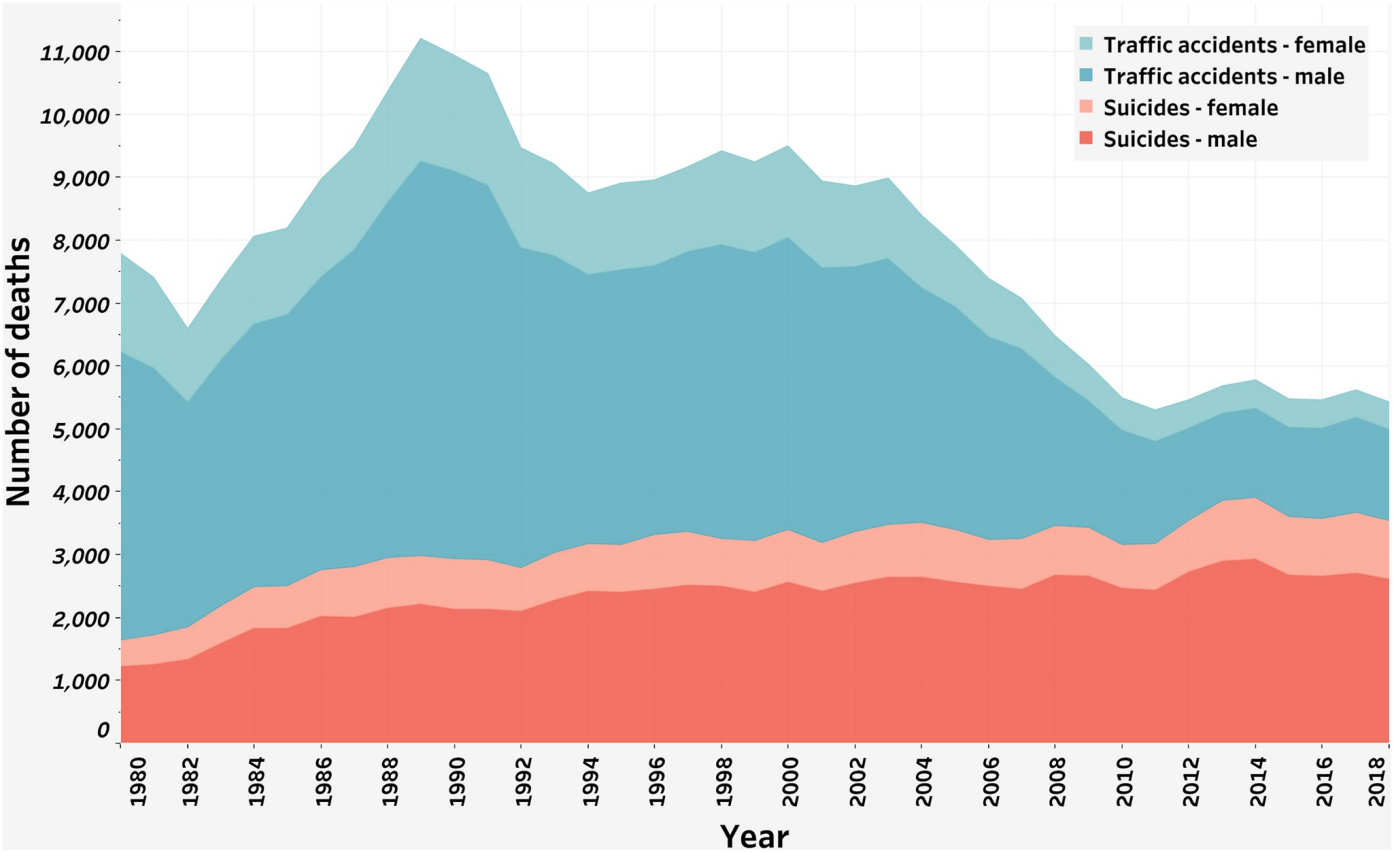
Evolution of deaths due to suicide and traffic accident by sex (1980–2018). *Source*: Death statistics according to cause of death from the Spanish Statistical Office (INE).

During the last decades, the Spanish deaths due to other external causes such as drug consumption or traffic accidents have been successfully prevented through several campaigns. For instance, the Spanish government implemented national strategic plans to deal with traffic accidents in 1980 (Plan Nacional de Seguridad Vial) and drug consumption in 1985 (Plan Nacional sobre Drogas). However, Spain has experienced a gradual, but steady, increase in suicides over the years, given the lack of an effective suicide strategy plan. Spain has historically integrated suicide prevention into their national mental health plan (Estrategia en Salud Mental del Sistema Nacional de Salud). In recent years, suicide prevention has been considered by healthcare professionals and policy-makers as a main priority to deal with in following mental health strategic plans. As we can see in Fig 1, suicides are the current leading external cause of death and of particular concern for men, whereas traffic accidents have undergone a substantial and constant decrease over the years.

On the other hand, global effort to address suicide has surged in recent years. In 2014, the WHO published a suicide prevention report: *Preventing suicide: a global imperative* [29], making a call to action for countries to adopt national suicide prevention strategies and suggesting the need for a multisectoral approach in health promotion. Steps for developing suicide prevention strategies followed and around 40 countries have developed or revised their national strategy. Moreover, attention on the matter was restated by WHO in 2018 by publishing another report aiming to support countries in continuing the progress in preventing suicide and to encourage establishing or revising national suicide prevention strategies: *National suicide prevention strategies: Progress, examples and indicators* [30]. It seems clear that the implementation of suicide prevention through action plans and defined national strategies is necessary to cope with a long-term neglected important health issue. Moreover, these prevention strategies have been recommended to focus on the population as a whole and vulnerable persons in particular. Therefore, understanding the mechanisms impacting on the vulnerability to suicidal behaviour as the aim of this study will help to boost the effectiveness of prevention policies and action plans taken in place.

**Fig 2:**
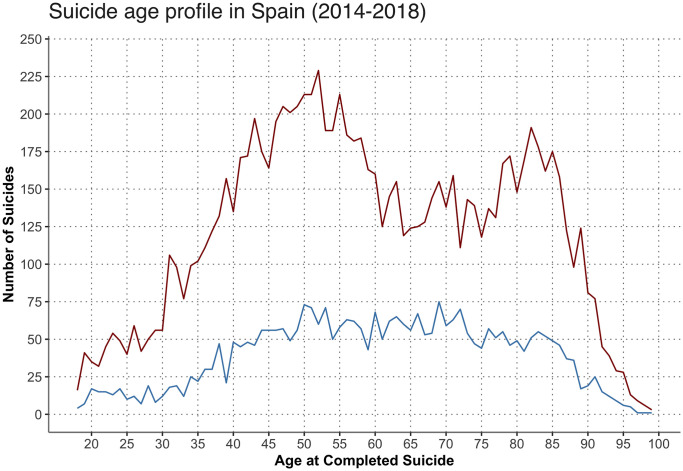
Number of suicides by age and gender between 2014–2018 (Spain). *Source*: *Deaths Statistics according to Cause of Death (INE)*.

Turning to Spain, although Spanish suicide rates are lower than most other developed countries, it is still an important cause of death in all age groups, and particularly concerning working-age people. Suicide rate increases with age, especially for men, and the number of completed suicides peaks for individuals 40–59 years old, age range comprising 41% of completed suicides in 2018, as it can be seen in Fig 2. However, youth suicide has also received plenty of worldwide attention as is one of the leading causes of death in adolescence and youth adulthood. In Spain, completed suicide is the second leading cause of death among young people but an important serious public health issue across all working-age groups.

Therefore, we consider that understanding and estimating the EPL impact on completed suicides is relevant for suicide prevention policies and of interest to healthcare institutions. In Spain, the National Health System is highly decentralized to the 17 Spanish Autonomous Communities, which are in charge of the management and provision of health care. While, the management and provision of health care of the two Autonomous Cities, Ceuta and Melilla, are in charge of the Spanish government. Among their functions, the regional governments are in charge of the design and implementation of health-related legislation. However, the Spanish Ministry of Health is responsible for the coordination of national plans, and therefore the development of effective prevention strategy plans will require a multi-actor collaboration at different administrative levels.

## 3 Data and empirical strategy

### 3.1 Data

We use the Death Registers conducted by the Spanish National Institute of Statistics (INE). Under an agreement with the INE, we were granted access to the restricted licence version of the *Death Statistics* which contains individual death data by the cause of death. This dataset registers all deaths occurred in Spain and, from 2012 onwards, includes individuals educational level attainment. We use data from all available years, 2012 to 2018, and differentiate between two periods depending on macroeconomic conditions. According to the *Encuesta Población Activa (EPA)*, the Spanish Labour Market started registering an increase of employed individuals from the first quarter of 2014 onwards, after six years of decreasing absolute numbers. For each death, the dataset contains personal information on the date of death, the country of birth, gender, year and month of birth, the Spanish Autonomous Community of birth and death, educational level attainment and the cause of death. In our sample, we select the cohorts born between 1964 and 1972 so individuals are aged 41–54 during 2012–2018. We exclude foreign-born from the data and, therefore, restrict the analysis to the Spanish native sample.

The information available on the cause of death follows the classification criteria by the 10th revision of the International Statistical Classification of Diseases and Related Health Problems, ICD-10 [31]. Moreover, INE provides a reduced list of groups of cause (CIE-10) aggregating by ICD-10 subdivisions. In our analysis, we use codes related to the external causes of death (90 to 102) of the INE reduced list CIE-10. Specifically, code 98 is used for Suicides (classified as X60-X84 in ICD-10), 95 for Drugs Abuse (classified as X41, X42, X44 and X45 in ICD-10) and 92, 93 and 97 are jointly used as a placebo test incorporating external deaths causes attributed to *accidental reasons*.

In order to obtain the mortality rates by educational level attainment groups, we combine the deaths dataset with the 2011 Census. The reference date of the Population Census is the 1st November 2011, therefore we assume the 1st January 2012 population to be similar to the reported in the 2011 Census. For each subsequent quarters, we extract the number of deaths that occurred during that particular quarter to the previous population by education, gender and quarterly cohort group. Then, we match the obtained populations to the corresponding deaths and calculate the quarterly cohorts mortality rates per 100.000 individuals.

The reform was fully implemented by October 1984 and the quarterly cohorts selected for our analysis depend on the individual educational level attainment and the corresponding age of labour market entry. In the case of high-school dropouts, an entry-age of 16 is considered, as the minimum working age in Spain is set at 16-years-old. However, first-level high school graduates and second-level high school graduates entering the labour market the last trimester of 1984, must have graduated in the summer of 1984. On one hand, first-level high school graduates are those who studied until the course corresponding to the 16 years of age (currently *ESO* in Spain). On the other hand, second-level high school graduates correspond to college-preparatory high school or basic Vocational and Technical Schools (*Bachillerato* and *CFGM*, respectively). Hence, the first trimester cohorts graduating in 1984 are considered as the entry-age to the labour market for these specific education groups. For instance, the cohort born in the first trimester of 1968 is the first cohort affected by the 1984 reform in the case of first-level high school graduates. In contrast, for second-level high school graduates, the first cohort affected is the cohort born in the first trimester of 1966.

Therefore, we have a total of 53,730 deaths recorded in the sample, 36,438 native males and 17,292 native females, who entered the labour market within four years before or after the 1984 reform could affect them. Of these recorded deaths, 33,017 individuals were born before they could be affected by the reform whereas 20,713 individuals after. A further description of the number of deaths by sex, educational attainment level and the causes of deaths can be found in Table 1.

**Table 1:** Sample summary.

	HS dropouts		HS 1st level grad		HS 2nd level grad	
	Male	Female	Male	Female	Male	Female
	(1)	(2)	(3)	(4)	(5)	(6)
Suicides	293	69	815	216	482	191
Drugs abuse	95	18	245	50	105	24
Accidental	192	47	464	62	225	60

*Source*: Death Statistics according to Cause of Deaths (INE) sample for years 2014–2018 of Spanish natives who are high school dropouts (born between 1964q4 and 1972q3), high school first-level graduates (born between 1964q1 and 1970q4) and high school second-level graduates (born between 1962q1 and 1969q4).

### 3.2 Empirical strategy

We analyze the effect of the 1984 labour reform on the long-term suicide mortality rates outcomes. The key explanatory variable is the trimester of birth cohort, which determines if those individuals entered the labour market before or after being affected by the liberalisation of fixed-term contracts. The cutoff on the trimester of birth depends on the education group. For high school dropouts, the cutoff is set as the fourth trimester of 1968. Therefore, treated cohorts are those who turn 16 from October to December in 1984 or after, while older cohorts are left untreated as they enter the labour market before the full implementation of the reform. As for first-level high school graduates, the trimester of birth cutoff is defined as the first trimester of 1968 due to their graduation date, whereas for second-level high school graduates is set at the first trimester of 1966. We measure the reform impact by estimating the jump in the suicide mortality rates for the cutoff cohort. It must be stated that the untreated cohorts are also affected by the labour reform some quarters after entering the labour market. However, García-Pérez et al. (2019) [12] argument that the impact on these cohorts is very small because the conditions at labour market entry are of particular relevance on long term career outcomes [32–33]. Moreover, they argue that there is a discontinuous jump in the availability and use of fixed-term contracts in Spain before and after the implementation of the reform.

As existing literature suggests that macroeconomic conditions have an impact on suicide risk, we restrict the analysis to an economic upswing period in Spain (2014–2018). Then, we collapse the individual-level data by the trimester of birth control (c) and the year of death (t). We run a cohort regression discontinuity design [34], including different trends on each side of the cutoff, and add the years of death as a fixed effect to capture macro-level factors:

$$\begin{array}{c} Y_{ct}=\beta_{0}+\beta_{1}\;\left(BirthTrim_{c}-C\right)+\beta_{2}\;\left(BirthTrim_{c}-C\right)*after_{c}+\beta_{3}\;after_{c}+\phi_{t}+\epsilon_{ct} \end{array}$$ (1)

where C is the treatment cut-off set at the first cohorts affected by the reform. *Y*_*ct*_ is the cause-specific mortality rate per 100,000 individuals for each cohort and year of death. A linear cohort trend is included by subtracting the cutoff from the cohort’s birth trimester (*BirthTrim*_*c*_ − *C*). Then, we interact the trend with a dummy variable (*after*_*c*_) that captures the cohorts affected by the reform, allowing us to include a different trend only for these cohorts. *ϕ*_*t*_ is a year of death fixed effect and *ϵ*_*ct*_ is the error term. Standard errors are clustered by cohort.

The impact of the reform is captured by the *after*_*c*_ variable, a binary variable equal to 1 for cohorts born at the cutoff trimester or after, and equal to 0 for cohorts born before. Therefore, the coefficient of interest is *β*_3_, which estimates the differential effect of the reform in the cause-specific mortality rates for the treated and untreated groups. Under the assumption of continuity around both sides of the reform cutoff point, *β*_3_ measures the long-term causal effect of the liberalisation of fixed-term contracts on cause-specific mortality rates (i.e., suicide rates). This key identification assumption was already addressed by García-Pérez et al. (2019) [12] through a differences-in-differences approach for labour market outcomes, and they also provide a descriptive analysis of the running variable by education. We also address it through two placebo test in Section 4.

The bandwidth used for the regression discontinuity analysis is selected to include 16-trimester cohorts before and 16-trimester cohorts after the cutoff. While a larger bandwidth yields more precise estimates, since more cohorts are used, it can lead to a biased estimation of the treatment effect. Therefore, this concern is addressed by reducing the bandwidth in Table 3. Specifically, we report a bandwidths of 6 and 10 cohorts respectively, the former being selected through the data-driven bandwidth point estimator proposed by Calonico et al. (2017) [35]. The larger results we find due to reducing the bandwidth are indicative that the possible bias in the 16 cohort-bandwidth is towards zero. Therefore we report both results in the discussion and the conclusion. The number of bins is limited by the size of the bandwidth as trimester cohorts bins are chosen for a more straightforward interpretation. As a result of the low number of cohorts (32 cohorts), we calculate the standard errors using the Wild Bootstrapping method proposed in Cameron et al. (2008) [36] and p-values are reported in brackets.

## 4 Results

Before focusing on our analysis of the long-term impact of the liberalization of fixed-term contracts on suicide, we rely on the estimates of García-Pérez et al. (2019) [12] on the impact of the reform on the number of temporary contracts. In their paper, the authors analyse the impact of the reform on the annual number of fixed-term contracts and all non-permanents contracts held by male high-school dropouts and find a strong increase by 14.6% of fixed-term contracts and by 17.7% of non-permanent contracts per year. Therefore, entering the labour market after the reform increased the probability of holding a fixed-term contract in the long run for less educated workers. This large long-term effect suggests that the reform led a group of less educated workers on a worse career path without upgrading from temporary employment to a permanent contract. It is thus consistent with the model by Blanchard and Landier (2002) [11]. Their theoretical model points out that the coexistence of fixed-term and permanent contracts can offset the gains of flexibilisation. While the liberalisation and reduction of firing costs of fixed-term contracts induce firms to contract more entry-level workers, it lowers the bargaining power of temporary workers who can easily get fired. As the firing costs in permanent jobs are left unchanged, firms are less prone to upgrade temporary employees to permanent contracts. As a result, low productivity jobs are recurrently filled with fixed-term contracts. Moreover, employers incentives to invest on training workers differ between those under temporary and permanent contracts. As this training investments develops specific-firm, but also general skills, those that enter the labour market under a permanent contract enjoy a positive skill gap with respect to those with temporary contracts. Therefore, less educated workers entering the labour market after the fixed-term contract liberalisation are put on a worse career path compared to the prior cohorts.

García-Pérez et al. (2019) [12] also show that the negative labor market effects for the affected cohorts take some years to materialize. Therefore, as the negative labor market effects are accumulating and growing over time, we believe that the impact on the mental health situation of those workers will also take some time to show up. Given these evidence on the labour market effects in the long-run, in our empirical work we focus on the long-term side effects of the labour market reform on suicide rates.

**Fig 3:**
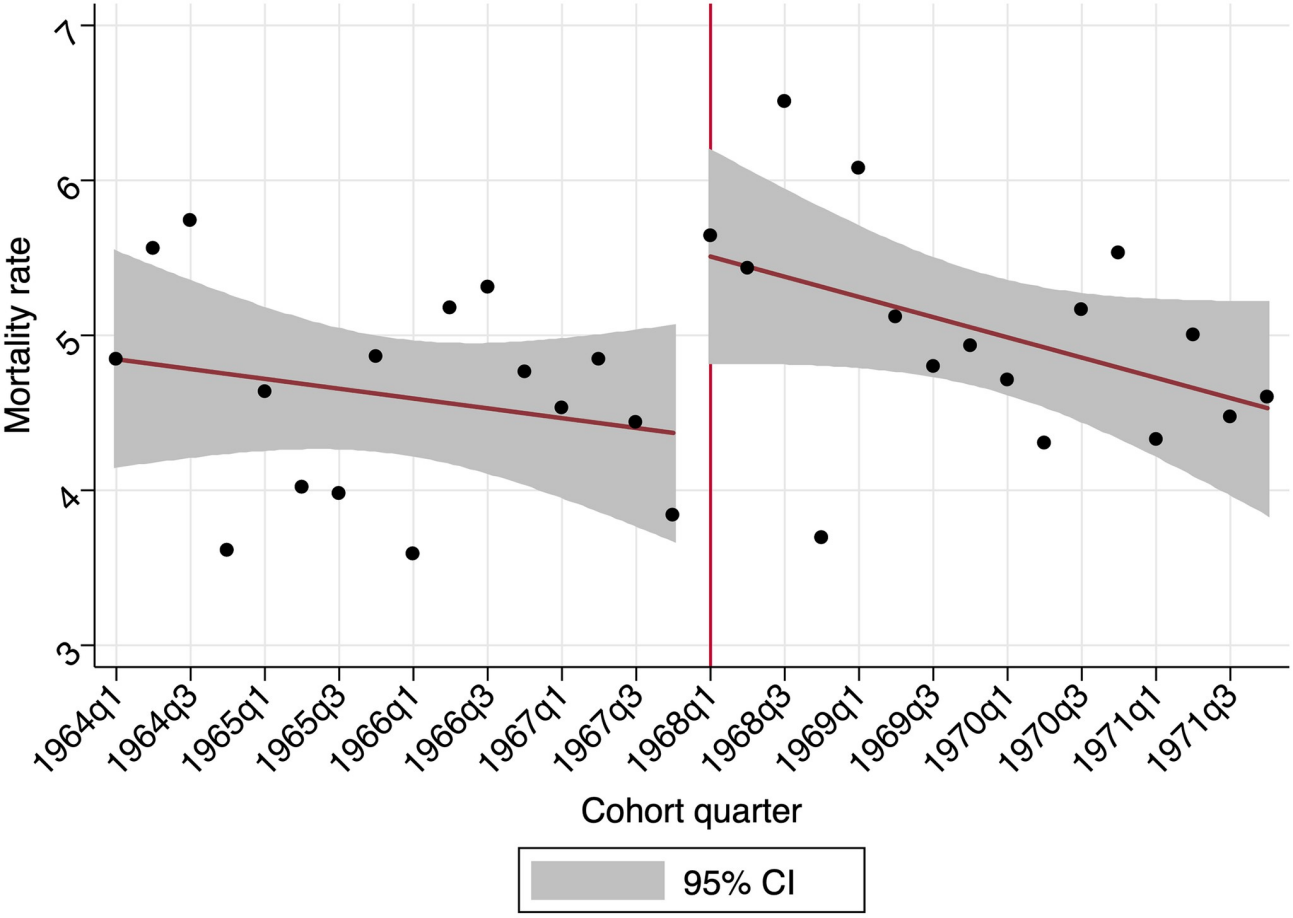
Suicide mortality rates—Spain-born high school 1st level male grad (2014–2018). The figure shows the evolution of the quarterly suicide mortality rate as deaths per 100000 individuals during 2014–2018 for Spain-born males. The vertical line indicates the first cohort (1968q1) affected by the 1984 labour reform for high school 1st level graduates. *Source*: Death Statistics according to Cause of Deaths and the 2011 Census from the Spanish Statistical Office (INE).

To that end, we differentiate between different groups, which allows us to use the cohort regression discontinuity design of Eq (1) to estimate the causal effect of the reform on each of the sub-groups. To provide preliminary descriptive evidence on the effects of the reform, Fig 3 shows the evolution of the suicide mortality rates for the high school first level male graduate cohorts entering the labour market before and after (indicated with a vertical line) the liberalisation of fixed-term contracts during our period of analysis, 2014–2018. The figure shows quarterly suicide mortality rates expressed as deaths per 100,000 individuals and a linear fit model. It provides preliminary evidence that the reform fostered an increase in the suicide mortality rate of cohorts entering the labour market after its implementation. Particularly, a significant and positive coefficient on *after* for high school first level male graduates is estimated by the jump in suicide mortality rates at the cutoff trimester cohort.

S1 Fig in the Appendix section shows the same graphs for high school dropouts and high school second level graduates. However, there is no graphical evidence on the effects of the reform for these educational groups. Furthermore, S3 Fig provides the suicide mortality patterns for females in the three analysed educational attainment level groups and, again, there is no observable effect of the reform.

As mentioned, Table 2 presents the results of the estimation of suicide mortality rates for different educational levels and gender during 2014 to 2018. First, we estimate the specification including all individuals and controlling for *women* (columns (1), (4) and (7)). Then, we report the results separated by gender. Moreover, high school dropouts, born between the fourth trimester in 1964 and the third trimester in 1972, are reported in Columns (1) to (3). The estimations for high school first level graduates, born between the first trimester in 1964 and the fourth trimester in 1971, are reported in Columns (4) to (6). Finally, estimations for high school second level graduates, born between the first trimester in 1962 and the fourth trimester in 1969, are reported in Columns (7) to (9).

**Table 2:** Suicide mortality rates (2014–2018).

	High-school dropouts			High-school 1st level grad			High-school 2nd level grad		
	Both	Male	Female	Both	Male	Female	Both	Male	Female
	(1)	(2)	(3)	(4)	(5)	(6)	(7)	(8)	(9)
**A** **f** **t** **e** **r**	-0.183	-0.407	0.0399	0.607 ***	1.167 ***	0.0477	-0.0648	0.217	-0.347
	[1]	[0.96]	[0.92]	[<0.01]]	[<0.01]]	[0.68]	[0.96]	[0.8]	[0.6]
*Trend*	-0.027	-0.0056	-0.0492	-0.0164	-0.0312	-0.0015	0.0184	-0.0324	0.0691
	[0.6]	[0.88]	[0.16]	[0.2]	[0.24]	[0.8]	[0.56]	[0.56]	[0.28]
*Trend* × *After*	-0.0134	-0.0716	0.0447	-0.0292	-0.0340	-0.0243	-0.0417	0.0204	-0.104
	[0.92]	[0.72]	[0.72]	[0.36]	[0.56]	[0.44]	[0.36]	[0.6]	[0.16]
*Women*	-3.402 **			-3.430 **			-2.692 **		
	[0.04]			[0.04]			[0.04]		
*Constant*	4.453 ***	2.577 ***	0.975	4.372 ***	4.505 ***	2.012 ***	4.112 ***	3.927 ***	2.831 ***
	[<0.01]	[<0.01]	[0.16]	[<0.01]	[<0.01]	[<0.01]	[<0.01]	[<0.01]	[<0.01]
Quarter FE	Yes	Yes	Yes	Yes	Yes	Yes	Yes	Yes	Yes
Cluster	Cohort	Cohort	Cohort	Cohort	Cohort	Cohort	Cohort	Cohort	Cohort
Observations	1280	640	640	1280	640	640	1280	640	640
$R_{Adj}^{2}$	0.118	0.082	0.031	0.225	0.0605	0.0366	0.100	0.0400	0.0179
Pre-Reform mr	3.43	5.29	1.57	3.04	4.61	1.46	3.14	4.52	1.76

* p <0.1,

** p <0.05,

*** p <0.01.

*Notes*: Robust standard errors are clustered at the birth cohort level and calculated using the wild bootstrapping method proposed in Cameron et al. (2008), p-values are reported [in brakets]. FE by quarter of death. Quarterly mortality rates reported as deaths per 100000 population.

*Source*: Death statistics according to cause of death (INE) sample of Spanish natives who are high school dropouts (born between 1964q4 and 1974q3) in Columns 1 to 3, high school first-level graduates (born between 1964q1 and 1970q4) in Columns 4 to 6 and high school second-level graduates (born between 1962q1 and 1969q4) in Columns 7 to 9, and population data from the Spanish Census 2011.

We find that the reform increased the quarterly suicide mortality rate for high school first-level graduates males at conventional significance levels (column (5)). This effect corresponds to a 25.3% increase on the suicide rates in the long run for those cohorts that entered the labour market after the liberalization of fixed-term contracts. The fact that the effect is driven by males is supportive of previous literature finding that employment instability particularly affects men’s health outcomes. This large impact on suicides is consistent with the findings that less educated cohorts entering the labour market under lax fixed-term contract legislation had substantially worse long-term labour outcomes in the long run.

The main channel through which the suicide rates increases is by changing the nature of the first contract when entering the labour market. Existing literature highlights the strong impact of the first employment experience on the future career. The liberalisation in the regulation of fixed-term contracts affected especially the first contract for less educated young workers while the effect on higher educated workers was not significant. This can be further related to the different incentives employers have to invest on training workers under a permanent contract respect to those under a temporary contract. Thus, we believe that this provides evidence that one of the main channels that explain the large impact on suicide rates in column (5) is the change in the first type of contract when entering the labour market which, in turn, leads to strong negative labour outcomes in the long run.

Furthermore, the *after* coefficient is not significant for high school dropouts (columns (1) to (3)) or for high school second level graduates (columns (7) to (9)). The latter is expected due to long-term labour outcomes of this higher education group not being particularly affected by the reform. However, high school dropouts were strongly affected by the liberalisation of fixed-term contracts, hence the lack of an impact on suicide rates may indicate different types of mechanisms among different educational groups. For example, it is possible that high school dropouts with tendency to commit suicide had particularly high suicide hazard rates before the period for which the data is available, specially during economic recessions. In this case, the subsample of dropouts during our sample period would be less affected by the reform (as they would have been impacted before) and we would not find a significant effect. Moreover, a larger impact of the reform could be expected on high school first level graduates as, according to the literature, suicides due to career issues are more common for this education group than for high school dropouts. Nevertheless, it is important to understand and disentangle the channels through which the liberalisation of fixed-term contracts affect less educated workers and different education groups, which is outside the scope of this paper.

We also report in S1 Table the specifications for a late-period of economic recession in Spain from 2012 to 2013. The preliminary descriptive evidence for this period is found in S2 Fig for males. As mentioned, one of the main challenges arising in suicide research is the low base rate of deaths. We consider that the number of suicides observed in some education group cohorts during this limited period is too low and consequently, these estimations are inconclusive for an economic recession period. Moreover, the estimates for these years could be particularly affected by higher suicide rates during the previous years (for which data is not available) when unemployment rates suddenly increased a lot in Spain.

## 5 Robustness checks and extensions

### 5.1 Robustness tests

Our identification strategy, based on a cohort regression discontinuity design, identifies the causal effects conditional on some assumptions that we test in this section. More specifically, the issues that we address in this section are: (i) the right size of the bandwidth, (ii) the functional form, and (iii) the continuity assumption at the cutoff point.

In our baseline results, we include 16-trimester cohorts before and 16-trimester cohorts after the implementation of the reform. As a robustness test, we check for the potential risk of including observations far from the cutoff point by reducing the bandwidth. In this nonparametric method, we limit the analysis to a closer surrounding of the cutoff, where local linear regressions are more likely, and estimate the treatment effect as local randomization. We reduce the bandwidth to 10-trimester cohorts and 6-trimester cohorts as shown in Table 3. Specifically, the later being selected through the data-driven bandwidth point estimator proposed by Calonico et al. (2017) [35].

**Table 3:** Robustness checks—Suicide mortality rates (2014–2018).

	10 trim. bandwidth	6 trim. bandwidth	2nd polynomial	3rd polynomial
	Male	Male	Male	Male
	(1)	(2)	(3)	(4)
**A** **f** **t** **e** **r**	1.218 ***	1.844 ***	1.115 *	2.379 ***
	[<0.01]	[<0.01]	[0.08]	[<0.01]
*Trend*	-0.002	-0.229 **	0.0436	-0.662 *
	[0.92]	[0.04]	[0.84]	[0.08]
*Trend* × *After*	-0.135	-0.0974	-0.183	0.411
	[0.08]	[0.16]	[0.28]	[0.2]
*Trend*^2^			0.004	-0.096
			[0.6]	[0.12]
*Trend*^2^×*After*			0.0001	0.120
			[1]	[0.12]
*Trend*^3^				-0.004 *
				[0.08]
*Trend*^3^ × *After*				0.003
				[0.12]
*Constant*	4.112 ***	3.246 ***	4.729 ***	3.581 ***
	[<0.01]	[<0.01]	[<0.01]	[<0.01]
Quarter FE	Yes	Yes	Yes	Yes
Cluster	Cohort	Cohort	Cohort	Cohort
Observations	400	240	640	640
$R_{Adj}^{2}$	0.0872	0.171	0.0609	0.0633
Pre-Reform mr	4.56	4.41	4.61	4.61

* p <0.1,

** p <0.05,

*** p <0.01.

*Notes*: Robust standard errors are clustered at the cohort level and calculated using the wild bootstrapping method proposed in Cameron et al. (2008), p-values are reported [in brakets]. FE by quarter of death. Quarterly mortality rates reported as deaths per 100000 population. Using the Calonico et al. (2017) data-driven bandwidth selection algorithm “rdbwselect”, it has been point estimated a bandwidth of 6 cohorts (column 2).

*Source*: Death Statistics according to Cause of Deaths (INE) sample of Spanish natives who are high school male first-level graduates, born between 1965q3 and 1970q2 (10 trimestres bandwidth), between 1966q3 and 1969q2 (6 trimestres bandwidth) and born between 1964q1 and 1971q4 (2nd and 3rd polynomial), and population data from the Spanish Census 2011.

In both cases, the impact of the reform on suicide rates is positive and of a similar size for males. The *after* coefficient for the 10 trimester-bandwidth estimates a 26.7% increase in the suicides rates (column (1)). Alternatively, the *after* coefficient for the 6 trimester-bandwidth estimates a 41.8% increase in the suicides rates (column (2)). Although all estimates indicate a large impact of the liberalisation in the regulation of fixed-term contracts on the suicide rates in the long run, the stronger impact shown when the 6 trimester-bandwidth is used reinforces the causal interpretation. While the rectangular kernel used in our RDD has a more straightforward interpretation, in the light of the results of the robustness test carried out, a triangular kernel weighting could be considered a good model fit too. The results of this first robustness test allows us to conclude that the large impact of the liberalisation in the regulation of fixed-term contracts on suicide rates is robust to bandwidth selection and that the impact ranges between an increase of 25.3% and a higher bound of 41.8%.

We next focus on the functional form of the *trend* variable used in the main results specifications. In our analysis, we use linear trends to estimate the local average treatment effect on suicides around the cutoff point. As a robustness test, we test different functional forms for the *trend* variable to minimize specification bias from the possibility of an incorrect linear functional form, which are reported in Table 3. Specifically, we use a parametric strategy changing the order of the polynomials to a second and third degree for trimester cohort trends. The different orders of the *trend* variable are interacted with the *after* dummy variable. Thus, the terms of the polynomial (slopes) estimated below the threshold are allowed to be different from the slopes estimated above the cutoff point.

Table 3 shows our baseline model specification using second and third degree polynomials for the trimester cohorts *trend* variable. As can be seen in the table, the large impact of the liberalisation in the regulation of fixed-term contracts on male suicide rates is robust to the inclusion of higher order polynomial trends (columns (3) and (4)). However, we choose the linear regression as our baseline model because it reports the minimum Akaike Information Criteria (AIC) among all the estimated models. Moreover, the second polynomial specification reports similar results than the linear fit.

It should also be noticed that our empirical strategy addresses possible anticipation effects. The last graduate untreated cohorts were born in the last quarter of the year and had between nine and twelve months to look for a permanent job before the reform was implemented on October 17th 1984. Whereas the first graduate treated cohorts were born in the first quarter of the year but were not able to work as they were attending school until the summer before the reform. In the summer of 1984 the reform had already been passed while traditionally being a season reporting high rates of *seasonal* temporary employment, which was already allowed before the reform. Therefore, concerns of spillover effects to untreated are severely mitigated.

Finally, we also perform placebo tests in order to address concerns on the identification assumption and, hence, to check the random assignment around the threshold.

We simulate the implementation of the reform to take place in a pre-reform period (4 years before) in Table 4. This allows us to examine if discontinuities are present at a different unexpected point of the assignment variable. As expected, the local estimates are insignificant and the placebo reform does not show an impact for the period analysed. While other fake thresholds can be checked, we consider this falsification test and the descriptive corroboration from Fig 3 to be evidence of the lack of discontinuities at other trimester cohorts.

**Table 4:** Falsification test: Pre-reform four years—Suicide mortality rates (2014–2018).

	Both	Male	Female
	(1)	(2)	(3)
**A** **f** **t** **e** **r**	-0.0106	-0.389	0.177
	[0.84]	[0.84]	[0.44]
*Trend*	0.0432	0.0757	0.0107
	[0.24]	[0.32]	[0.4]
*trend* × *after*	-0.0596	-0.107	-0.0122
	[0.16]	[0.28]	[0.68]
*Women*	-3.261 **		
	[0.04]		
*Constant*	4.687 ***	5.461 ***	2.482 ***
	[<0.01]	[<0.01]	[<0.01]
Quarter FE	Yes	Yes	Yes
Cluster	Cohort	Cohort	Cohort
Observations	1280	640	640
*R*^2^	0.213	0.0578	0.0549
Pre-Reform mr	2.89	4.59	1.21

* p <0.1,

** p <0.05,

*** p <0.01.

*Notes*: Robust standard errors are clustered at the cohort level and calculated using the wild bootstrapping method proposed in Cameron et al. (2008), p-values are reported [in brakets]. FE by quarter of death. Quarterly mortality rates reported as deaths per 100000 population. Placebo reform settled 4 year prior the actual reform, first cohort affected 1964q1.

*Source*: Death Statistics according to Cause of Deaths (INE) sample of Spanish natives who are high school first-level graduates (born between 1960q1 and 1967q4) and population data from the Spanish Census 2011.

Furthermore, as a second placebo test, we check whether other outcomes that are unlikely related to the reform are affected at the cutoff point. We consider deaths due to external accidents as an ideal candidate for the placebo test. Specifically, the placebo outcome comprises numbers 92, 93 and 97 of the INE reduced list CIE-10. They consist of falls, accidental drowning and submersion, other accidental threats to breathing and other types of unclassified accidents. These accidents are unlikely to be affected by the reform as INE list also includes specific codes for accidents related to drugs, alcohol, mental health or traffic, which we do not included in the placebo test. Table 5 reports the estimates for the placebo test on the alternative outcome. The impact of the reform on the placebo outcome is insignificant for all specifications including the educational group clearly affected by the reform (column (5)).

These placebo tests reinforce our conclusion that the liberalisation in the regulation of fixed-term contracts had a large causal impact on the suicide rates of high school first-level male graduates in the long run.

**Table 5:** Placebo test: External causes due to accidental mortality rates (2014–2018).

	High-school dropouts			High-school 1st level grad			High-school 2nd level grad		
	Both	Male	Female	Both	Male	Female	Both	Male	Female
	(1)	(2)	(3)	(4)	(5)	(6)	(7)	(8)	(9)
**A** **f** **t** **e** **r**	0.467	0.489	0.446	0.0869	-0.0395	0.213	0.351	0.411	0.292
	[0.2]	[0.48]	[0.32]	[0.84]	[0.88]	[0.44]	[0.36]	[0.4]	[0.48]
*Trend*	-0.0141	-0.0143	-0.0140	-0.0229	-0.0280	-0.0177	-0.0005	-0.0022	0.0032
	[0.44]	[1]	[0.56]	[0.4]	[0.76]	[0.52]	[0.88]	[1]	[0.8]
*Trend* × *After*	0.0106	0.0140	0.00722	0.0130	0.0121	0.0139	-0.0595	-0.0743	-0.0447
	[0.92]	[0.88]	[0.92]	[0.56]	[0.72]	[0.52]	[0.24]	[0.24]	[0.36]
*Women*	-2.215 **			-2.341 **			-1.527 **		
	[0.04]			[0.04]			[0.04]		
*Constant*	2.739 ***	1.670	0.972	2.847 ***	3.163 ***	0.579 **	1.929 ***	2.688 ***	0.959 **
	[<0.01]	[0.16]	[0.2]	[<0.01]	[<0.01]	[0.04]	[<0.01]	[<0.01]	[0.04]
Quarter FE	Yes	Yes	Yes	Yes	Yes	Yes	Yes	Yes	Yes
Cluster	Cohort	Cohort	Cohort	Cohort	Cohort	Cohort	Cohort	Cohort	Cohort
Observations	1280	640	640	1280	640	640	1280	640	640
$R_{Adj}^{2}$	0.0608	0.0229	0.0384	0.213	0.0384	0.0326	0.0881	0.0314	0.0313
Pre-Reform mr	1.91	3.00	0.83	1.66	2.94	0.38	1.36	2.17	0.55

* p <0.1,

** p <0.05,

*** p <0.01.

*Notes*: Robust standard errors are clustered at the cohort level and calculated using the wild bootstrapping method proposed in Cameron et al. (2008), p-values are reported [in brakets]. FE by quarter of death. Quarterly mortality rates reported as deaths per 100000 population.

*Source*: Death Statistics according to Cause of Deaths (INE) sample of Spanish natives who are high school dropouts (born between 1964q4 and 1972q3) in Columns 1 to 3, high school first-level graduates (born between 1964q1 and 1971q4) in Columns 4 to 6 and high school second-level graduates (born between 1962q1 and 1969q4) in Columns 7 to 9, and population data from the Spanish Census 2011.

### 5.2 Drugs Abuse mortality

While the main focus of interest in our paper are suicides (as it is currently the leading external cause of death), the reform can have long-term effects on other cause-specific mortality rates as well. Therefore, in Table 6, we present a preliminary analysis of the mortality rates due to drug overdose, which can be linked to suicides.

**Table 6:** Drugs Abuse mortality rates (2014–2018).

	High-school dropouts			High-school 1st level grad			High-school 2nd level grad		
	Both	Male	Female	Both	Male	Female	Both	Male	Female
	(1)	(2)	(3)	(4)	(5)	(6)	(7)	(8)	(9)
**A** **f** **t** **e** **r**	-0.183	-0.151	0.541	0.406 **	0.793 **	0.0187	0.0923	-0.0391	0.224
	[1]	[0.88]	[0.4]	[0.04]	[0.04]	[0.8]	[0.56]	[1]	[0.24]
*Trend*	-0.0274	0.0911	-0.0064	0.00879	0.00379	0.0138	0.0130	0.0219	0.00415
	[0.6]	[0.2]	[0.8]	[0.48]	[1]	[0.16]	[0.64]	[0.68]	[0.8]
*Trend* × *After*	-0.0135	-0.140	0.00304	-0.0236	-0.0225	-0.0246	-0.0228	-0.0174	-0.0281
	[0.92]	[0.12]	[0.76]	[0.44]	[0.76]	[0.16]	[0.56]	[0.76]	[0.32]
*Women*	-3.402 **			-1.127 **			-0.748 **		
	[0.04]			[0.04]			[0.04]		
*Constant*	4.453 ***	1.219 *	0.243	1.430 ***	0.632	1.043 ***	1.067 ***	0.665	0.558
	[<0.01]	[0.08]	[0.8]	[<0.01]	[0.12]	[<0.01]	[<0.01]	[0.24]	[0.2]
Quarter FE	Yes	Yes	Yes	Yes	Yes	Yes	Yes	Yes	Yes
Cluster	Cohort	Cohort	Cohort	Cohort	Cohort	Cohort	Cohort	Cohort	Cohort
Observations	1280	640	640	1280	640	640	1280	640	640
$R_{Adj}^{2}$	0.118	0.0637	0.0396	0.107	0.0574	0.0352	0.0508	0.0225	0.0272
Pre-Reform mr	0.81	1.45	0.18	0.70	1.11	0.30	0.53	0.88	0.18

* p <0.1,

** p <0.05,

*** p <0.01.

*Notes*: Robust standard errors are clustered at the cohort level and calculated using the wild bootstrapping method proposed in Cameron et al. (2008), p-values are reported [in brakets]. FE by quarter of death. Quarterly mortality rates reported as deaths per 100000 population.

*Source*: Death Statistics according to Cause of Deaths (INE) sample of Spanish natives who are high school dropouts (born between 1964q4 and 1974q3) in Columns 1 to 3, high school first-level graduates (born between 1964q1 and 1970q4) in Columns 4 to 6 and high school second-level graduates (born between 1962q1 and 1969q4) in Columns 7 to 9, and population data from the Spanish Census 2011.

It is important to note that mortality rates due to drug overdose are much lower compared to suicides for the cohorts analysed. Moreover, there are large differences in drug consumption patterns between women and men, and the male mortality rate due to drug overdose is much higher than for women. In Spain, a national strategic plan to deal with drug consumption, Plan Nacional sobre Drogas, was adopted by the Spanish government in 1985. However, during the decade of the 1990s Spain register record-high drug overdose deaths because of a heroin epidemic, in which young male adults were a particularly susceptible target.

We estimate the same baseline specification than in Table 2 but changing the dependent variable to drug overdose quarterly mortality rates. Specifically, the analysed outcome coresponds to the code 95 of the INE reduced CIE-10 list (psychoactive substances and drug abuse), which is composed of following ICD-10 codes: X41, X42, X44, X45. Different specifications according to the educational attainment level and gender are reported as for suicides mortality rates. A further preliminary descriptive evidence can be found in S4 Fig.

The positive and significant *after* coefficient in column (5) indicates that there is a 71.4% jump in the mortality rate due to drug overdose of the high school first-level male graduates cohorts after the implementation of the liberalization reform. This is a really strong impact but it has to be noted that the number of deaths due to this cause is relatively small so that, potentially, a larger number of observations would be needed for a more precise estimation. In any case, it represent another indicator that the liberalisation in the regulation of fixed-term contracts had a negative impact on health outcomes in the long run. Thus, even if the specification estimates are less precise, it reinforces our main baseline results pointing towards the role of Employment Protection Legislation (EPL) on long-run health outcomes.

## 6 Conclusion

Suicide is a serious public health problem and one of the leading causes of death in the working-age population of developed countries. Our paper focuses on the estimation of the long-term effects of a reform that liberalised the use of temporary contracts on suicide rates. As argued by Blanchard and Landier (2002) [11], this type of reforms and the coexistence of fixed-term contracts with permanent ones, have a negative impact on long-term labour outcomes of less educated workers. For Spain, García-Pérez et al. (2019) [12] find that the long-term impact of the Spanish liberalization reform amounted to a 7.3% yearly earning losses for low skilled workers. This worse career paths brought about by the reform are expected to have side effects on health outcomes, which is what we determine and quantify in this paper.

We evaluate the impact on suicide mortality rates of the reform using a restricted version of the *Death Statistics* from the Spanish National Institute of Statistics (INE). We find that cohorts of male high school first-level graduates who entered the labour market after the reform have higher suicide rates in the long run compared to the cohorts that entered the labour market just before. Specifically, entering the labour market after the liberalisation of fixed-term contracts leads to at least a 25.3% increase in suicide rates in the long run. The main channel of this negative long-term effect is through a higher probability of having a temporary employment relationship as a first contract when entering the labour market after the reform and, hence, accumulating a worse career path for low skilled workers.

Thus, our findings indicate that a laxer regulation of fixed-term contracts in Spain increased the long-term suicide rates for low-skilled workers. We also find a large impact of the reform on drug overdose mortality rates, indicating that other health outcomes related to suicides are also affected by the reform. Overall, these results suggest that sudden changes in Employment Protection Legislation (EPL) can have large long-term effects on health outcomes, and particularly on suicide rates. Reasonably, this analysis addresses a question of high policy relevance for suicide prevention strategies.

One important limitation of this study is the number of years for which data is available with information on both the cause of death as well as the level of education. Therefore, there could be important effects in the suicide rate (particularly for high school dropouts) during the years between the time of the reform and our sample period that we are unable to observe which, in turn, could lead to survival bias in our estimates. However, we believe that, if this type of bias exists in our context, it would lead our estimates towards zero. Thus, we believe that our estimates reflect a lower bound of the true lasting effects of the reform that liberalized the use of temporary contracts on suicide rates.

Many questions remain open for future research. For instance, it is crucial to understand the conditions (e.g., family structures, job sectors or location) that reinforce the negative impact of the liberalisation reform, and for which specific sub-groups of the population. This would allow policymakers and healthcare professionals to identify potential vulnerable individuals to suicidal behaviour and boost the effectiveness of prevention policies and action plans adopted.

## Supporting information

S1 Table Suicide mortality rates (2012–2013). (TIF)

S1 Fig Evolution of Spain-born males suicide mortality rates (2014–2018). (TIF)

S2 Fig Evolution of Spain-born males suicide mortality rates (2012–2013). (TIF)

S3 Fig Evolution of Spain-born females suicide mortality rates (2014–2018). (TIF)

S4 Fig Evolution of Spain-born males mortality rates due to drug overdose (2014–2018). (TIF)
