# Correction: The impact of temporary contracts on suicide rates

**DOI:** 10.1371/journal.pone.0298372

**Published:** 2024-02-01

**Authors:** Ricard Grèbol Jiménez, Judit Vall Castelló

There is an error in the published funding statement. The correct funding statement is as follows: This project has received funding from Instituto de Salud Carlos III (reference of the project: AC19/00078) under the umbrella of the European Joint Programming Initiative “A Healthy Diet for a Healthy Life” (JPI HDHL) and of the ERA-NET Cofund HDHL INTIMIC (GA N° 727565 of the EU Horizon 2020 Research and Innovation Programme).
