# Supplementary figures and images for: The impact of temporary contracts on suicide rates
Source: PLoS One. 2021 May 26;16(5):e0252077. doi: 10.1371/journal.pone.0252077 (PMC8153446; doi:10.1371/journal.pone.0252077)

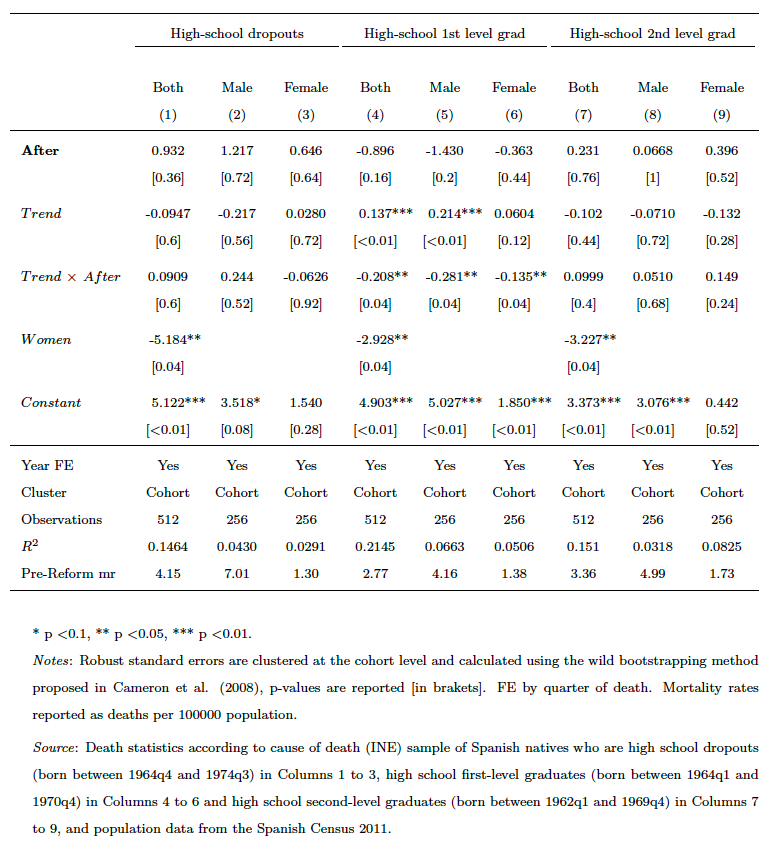

Supplement: S1 Table — (TIF) [file pone.0252077.s001.tif]

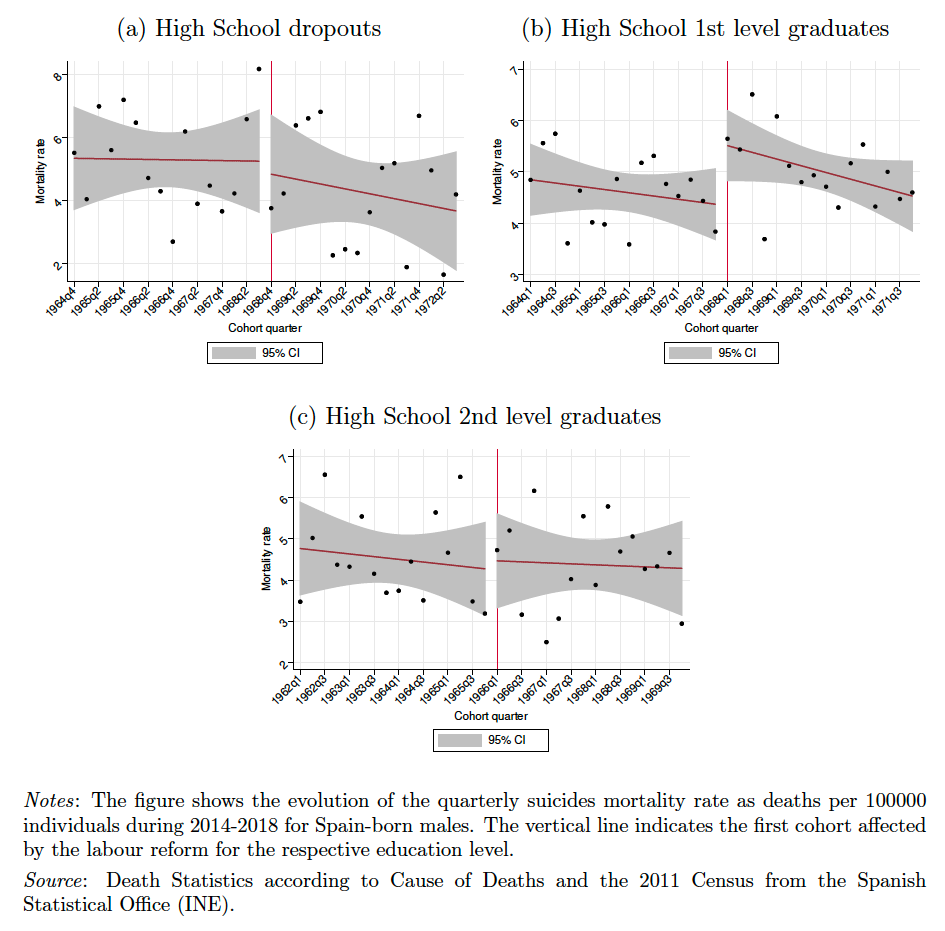

Supplement: S1 Fig — (TIF) [file pone.0252077.s002.tif]

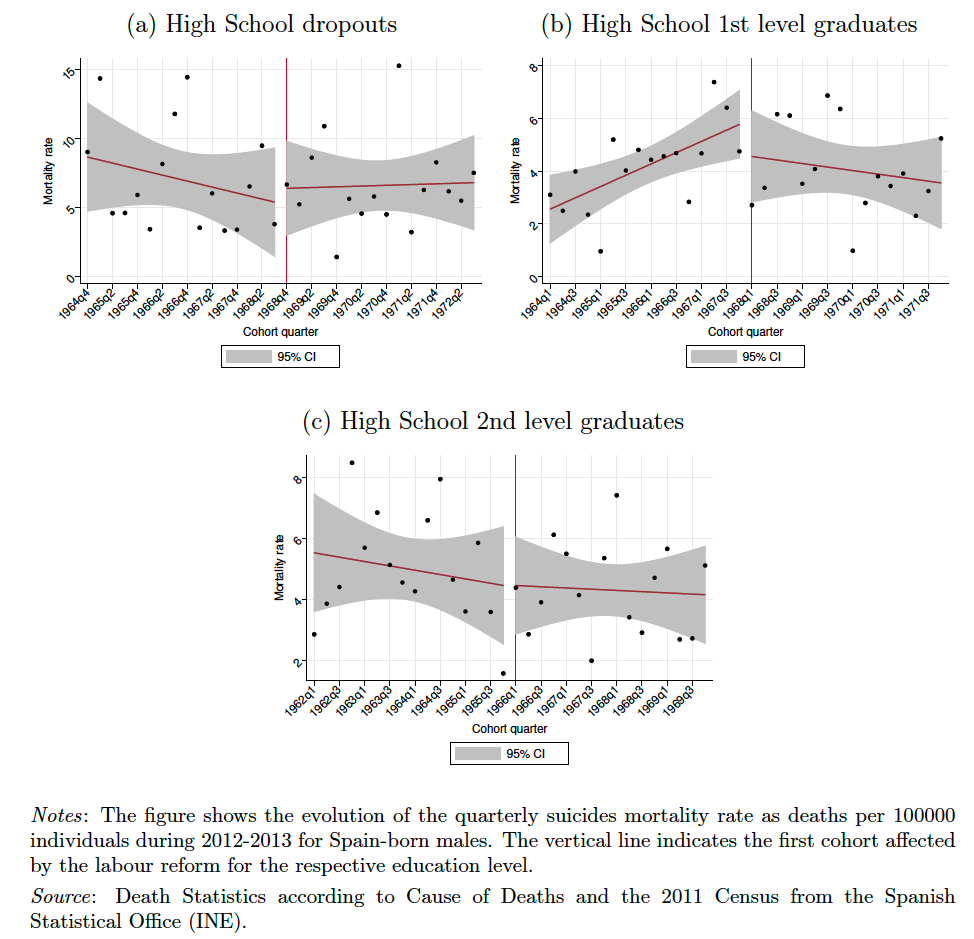

Supplement: S2 Fig — (TIF) [file pone.0252077.s003.tif]

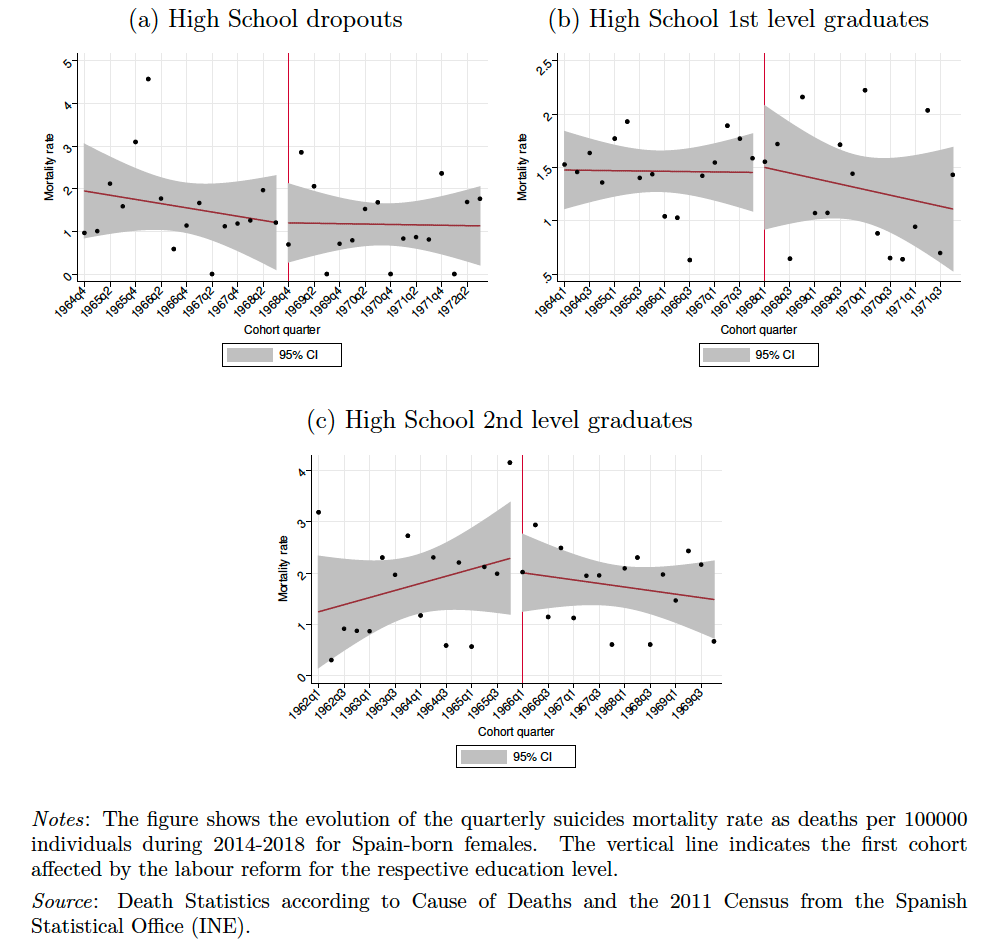

Supplement: S3 Fig — (TIF) [file pone.0252077.s004.tif]

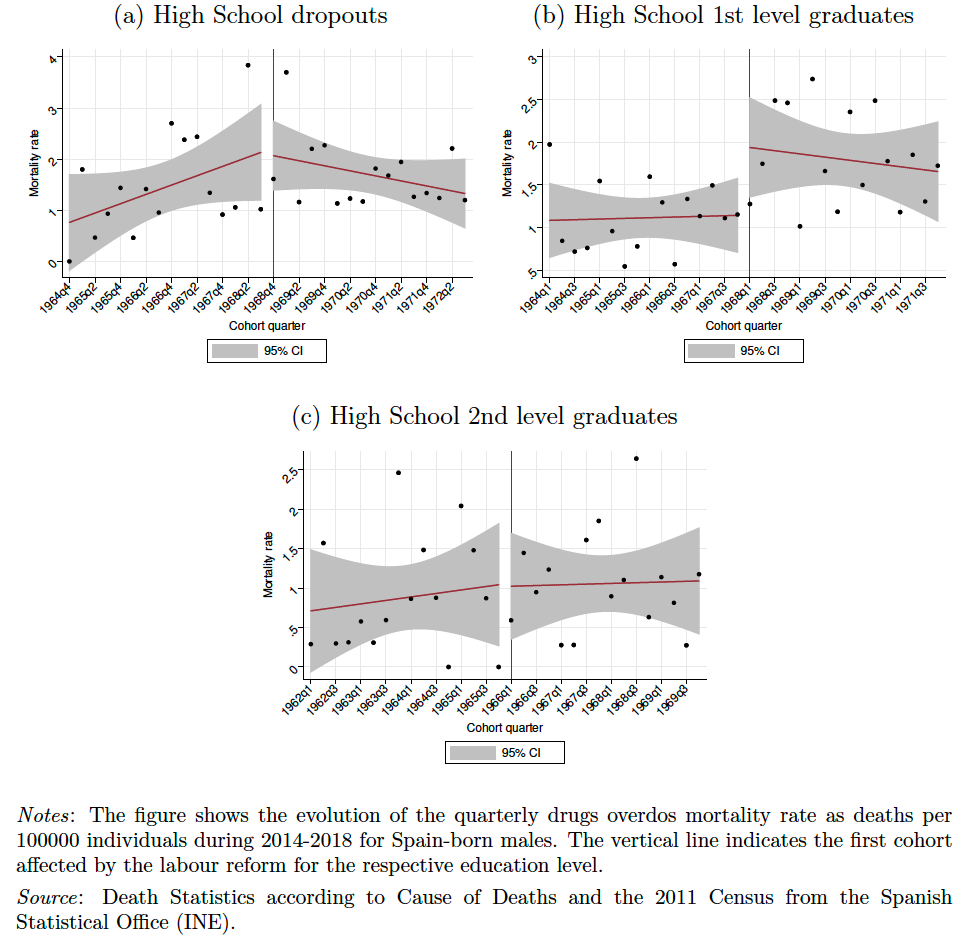

Supplement: S4 Fig — (TIF) [file pone.0252077.s005.tif]
